# The Chemist’s Dream

**Published:** 1853-02

**Authors:** 


*The Chemist's Dream.*

[The following ingenious and singular effusion has been placed in our hands by a chemical friend. We do not vouch that it has never been published before, but it will repay perusal during an idle moment. We are informed that it was actually written by a medical student some years ago in Philadelphia, but of the exact time of writing, or of the name of the author, the deponent saith not.—*Stethescope.*]

Methought I was exploring the hidden recesses of an extensive cave, whose winding passages had never before echoed to the tread of human foot. With admiration and delight I was gazing at the thousand wonders which the flashing torchlight revealed on every side, at each step of my progress, when a strange sound, as of the hum of many voices, fell upon my ear. What such a sound could mean, in such a place, was more than I could divine. Curiosity led me in the direction whence it came. The buzz of conversation, cheerful as it would seem from the occasional bursts of merriment that were heard, grew more and more distinct, until the dark and narrow passage I had been following suddenly opened upon one of those magnificent rock parlors, of whose grandeur and beauty description can convey but a faint idea. A flood of light illuminated the arching roof, with the vast columns of stalactites sparkling with crystals that supported it, and was reflected with imposing effect from the huge streets of the same material, of the purest white, that hung from the ceiling in graceful but substantial drapery. I stood in one of nature's noblest halls, but not alone.

A strange company had gathered there. Black spirits and white, blue spirits and gray, were before me. A festive occasion had assembled, in joyous mood and holiday attire, the first-born of creation—the *Elements* of things.

In dreams, nothing ever surprises us. It seemed perfectly natural to see these fairy forms in that grotto. So, accosting without hesitation the one nearest to me, I apologised for my intrusion, and was about to withdraw. From my new acquaintance, however, I received so cordial a welcome and so earnest an invitation to become a participator in their festivities, that I could not deny myself the pleasure of accepting the hospitality so kindly offered.

I was soon informed that some of the leading characters among the elements had resolved, some weeks previous, upon having a general pic-nic dinner party. Sixty-three family invitations had accordingly been sent out—one to each of the brotherhood—and preparations for the feast made upon a most extensive scale. Sea and land had been ransacked for delicacies, and everything was put in requisition that could contribute to the splendor of the entertainment or the enjoyment of the occasion.

At the hour I so unexpectedly came upon them, nearly all the guests, with their families, had assembled in the strange drawing-room I have described, awaiting the summons to the banquet.

Spacious as the drawing-room was, it was nearly filled with these interesting children of nature. And here they were seen, not as in the chemist's laboratory, writhing in the heated crucible, or pent up in glassy prisons, or peering out of gas-holders and Florence flasks, but arrayed in their beauty, each free as air, and acting as impulse prompted. There were those present of every hue, every style of dress, every variety of appearance. The metals, the gases, the salts, the acids, the oxides, and the alkalies—all were there.

From the mine, from the shop of the artizan, from the mint, from the depths of ocean even, they had come; and a gayer assemblage, a more animating scene, my eyes had never beheld. Many of the ladies of the party were most tastefully attired.

*Chlorine* wore a beautiful greenish yellow robe, that displayed her queen-like figure to good advantage. The fair daughters of *Chromium* particularly attracted my attention, with their gay dresses of the loveliest golden yellow and orange red. *Iodine* had just arrived, and was not yet disencumbered of an unpretending outer garment of steel grey that enveloped her person; but the warmth of the apartment soon compelled her to throw this aside, when she appeared arrayed in a vesture of thin gauze of the most splendid violet color imaginable. *Carbonic Acid* was there, but not clad in the airy robes in which I expected to see her. The pressure of the iron hand of adversity had been upon her, and now her attire was plain—simply a dress of snowy white—the best which the straitened circumstances to which she was reduced allowed her to assume. Quite a contrast to her was her mother, *Carbon*, whom you would have supposed to have been a widow in deep mourning, or a nun who had taken the black veil—so sable were her garments, so gloomy her countenance—had not her ear-rings of polished jet and a circlet of diamonds that glistened on her brow evinced that she had not altogether renounced the world and its vanities. The belle of the room appeared to be *Nitric Acid*, the graceful daughter of *Nitrogen*, airy in all her movements, and with dress of deepest crimson, that corresponded well with a lip and cheek rivaling the ruby in redness. Among the lady metals, too, there were many bright faces and resplendent charms; but I must pass on to a description of the gentlemen of the party.

*Sulphur* wore a suit of modest yellow plush, while *Phosphorus* quite disconcerted some of the most decorous matrons present, by making his appearance in a pair of flesh-colored tights.

*Phosphuretted Hydrogen*, or, as he is nicknamed, "Will-of-the-Wisp," startled me, by flitting by in a robe of living flame, the dress in which the graceless youngster is said to haunt church-yards and marshy places, playing his pranks upon poor benighted travellers. The king of metals, *Gold*, was arrayed in truly gorgeous apparel, though it must be confessed there was a glitter and an air of haughtiness about him, from which you would turn with pleasure to the mild, sweet face of his royal sister, *Silver*, who leaned upon his arm, a bright-eyed, unassuming creature of sterling worth.

*Mercury* was there, as lively and as versatile as ever, a most restless being, now by the thermometer, noting the subterranean temperature, now by the barometer predicting a storm in the regions overhead, now arm-in-arm with this metal, then with that, and they all, by the way, save stern old *Iron*, had hard work to shake him off. A strange character surely was he—a philosopher of uncommon powers of reflection—the veriest busy-body in the world, well versed in the healing art, a practical amalgamatist—in short, a complete factotum. *Potassium*, though a decidedly brilliant-looking fellow, manifested too much levity in his deportment to win respect, and was pronounced, by those who know him best, to be rather soft. In gravity, *Platinum* surpassed all the rest, and in natural brightness was outshone by few. When *Oxygen* arrived, and his light, elastic tread was heard, and his clear transparent countenance was seen among them, a murmur of congratulation ran around the drawing-room, and involuntarily all assembled rose to meet him and do him homage. He was a patriarch indeed among them—literally a father to many of the youngest guests. His arrival was a signal of adjournment to the banqueting-room, where of right he took his position at the head of the table.

Concerning the apartment we had now entered, I can only say it was grand beyond description. It was lighted up with the brilliance of noon-day by an arch of flame intensely dazzling, produced by a curious apparatus which *Galvanism*, who excels in these matters, had contrived for the occasion out of some materials which his friends *Zinc* and *Copper* had furnished him. Festoons of evergreens and wreaths of roses encircled the alabaster columns, and made the whole look like a hall in fairy-land. But I must describe the table and its paraphernalia—the preparation of viands, —I mean the baking, boiling, roasting, stewing, and the like, which had been committed to *Caloric*, who had long experience in that department. The nobler of the metals had generously lent their costly services of plate, while *Carbon* united with *Iron* to furnish the elegant steel cutlery used on the occasion. *Alumina* provided the fine set of china that graced the table, and *Silex* and *Potash*, without solicitation, sent as their joint contribution, cut glass pitchers and tumblers of superior pattern and transparency. As among the sons of nature there is no craving for artificial excitement, *Oxygen* and *Hydrogen*—who by the way have done more for the cold water societies than Delavan and Father Mathew—were commissioned to provide the drinkables, and what beverage they furnished may be easily conjectured. *Carbon*, with *Oxygen* and *Hydrogen*, found most of the vegetables, and *Nitrogen*, whose assistance as commissary here was indispensable, joined them in procuring the meats under which the table groaned. No taste but would be satisfied with variety—no appetite but would be cloyed with the profusion of good things. Though the liberality of the four that have been mentioned left but little for their associates to contribute, still some individual offerings to the feast deserve to be mentioned. Thus, the oysters, *Carbonate of Lime* had sent in shell; the pyramids of ice-cream for the dessert were provided by the daughter of *Chlorine* and *Hydrogen*, the bride of *Sodium*. who was out several hours in the snow engaged in freezing them; and the almonds and peaches came from the conservatory of *Hydrocyanic Acid*, the druggist.

After grace had been said by *Affinity*, who is a sort of chaplain to the *Elements*, having officiated at the weddings of all the married ones of the company, a vigorous onset was made upon all the good things before them. At first, all were too much engaged for conversation; but the dessert appearing at last, as they cracked their nuts, the jests too were cracked. Toast and song were called for, and wit and innocent hilarity became the order of the day. Even *Oxygen*, who had presided with such an air of dignity, relaxed from his sternness, and entertained the younger ones at the table with many a tale of his mischievous pranks in t e days of old father *Chaos*, when *Time* and himself were young. Strange tales they were, too, of earthquakes with which *Hydrogen* and he would now and then frighten the Ichthyosauri and Megatheria of the ancient world, and of conflagrations comical, as of old *Vulcan's* tongs and anvil, kindling them before his eyes with the very bolt he was forging. This, however, he added, with a sly glance at his old partner, *Nitrogen*, who sat near, was before marriage had sobered down his spirits and tamed his impetuosity.

I have no space to chronicle more of the freaks of *Oxygen's* early youth, nor any of the sayings and doings of others of this memorable night's party, else I might relate the marvellous story *Nickel* had to tell about the manner in which he managed to deceive and wrong the miners of former days, by making them believe that he was the parent of *Copper*, until at length they concluded that he was an evil spirit, whose sole object was to interrupt their operations. I would tell, too, of the drolleries of *Nitrous Oxide*, that funniest, queerest, craziest of youngsters, and how *Phosphorus* made a flaming speech, and *Potash* a caustic one, and how *Mercury* proposed as a toast, "The Medical Profession,—to whom we say, use us, but don't abuse us." I must speak, however, of a curious by-scene I chanced to witness. It was a flirtation that *Platinum* was carrying on with *Hydrogen*, whom, much to my surprise, I found seated up among the metals, and quite at home among them too. There was quite a contrast between *Platinum*, grey, heavy, and dull as he was, and the light and buoyant creature by his side, but there soon seemed to be evidence of some mutual attraction between them.

So passed the evening; all went on "merry as a marriage-bell," with nothing to mar the good humor that prevailed, until in an evil hour *Sulphuretted Hydrogen*, a disagreeable fellow, against whose appearance at the banquet most of the company had protested, entered the apartment with a very offensive air. In an instant the whole family of metals, to whom he is particularly obnoxious, changed color. *Lead* fairly grew black in the face with indignation; *Arsenic* and *Antimony* seemed to be jaundiced with rage; *Ammonia*, to whom his presence recalled very unpleasant associations, in trying to avoid him, precipitated several metallic oxides on the floor, while *Chlorine*, with more self-command than the rest, advanced with a firm step to expel the intruder, looking as if she were about to annihilate him on the spot. Well, at this crisis he spied *Nitric Acid;* and, knowing that his destruction was certain if they should come in contact, he at once withdrew, very much to the satisfaction of the whole company.

How the scene might have terminated I know not; for just at that moment a strange sound of awful import, like the trampling of a mighty host, came to my ears. I felt sure it was an earthquake's voice, and that now my fate was sealed. My knees tottered under me—the arching grotto and festive board gradually vanished from before my eyes, which opened upon the class as they were leaving the laboratory of our worthy professor of chemistry,—where, it seemed, much to my confusion, I had fallen asleep during the lecture, and

"Dreamed a dream in the midst of my slumbers."

